# A propos d’une observation de tumeur glomique de l’index

**DOI:** 10.11604/pamj.2017.26.155.11899

**Published:** 2017-03-15

**Authors:** Hicham Bousbaa, Laarbi Amhajji

**Affiliations:** 1 Department of Orthopaedics and Traumatology, Military Hospital Moulay Ismail, BP 50000 Meknes, Morocco

**Keywords:** Tumeur glomique, IRM, doigt, Glomus tumor, MRI, finger

## Image en médecine

La tumeur glomique (TG) individualisée par Masson est une tumeur bénigne développée aux dépend du glomus sous cutané. La glomus est l'organe de régularisation de la microcirculation capillaire et thermique. Elles représentent 1 à 5 % des tumeurs des parties molles de la main. La localisation est surtout sous unguéale (65%) mais aussi pulpaire et péri unguéale. Il s'agit d'une lésion d'évolution lente et de petite taille (en moyenne 3mm). La douleur est fulgurante spontanée et/ou provoquée par le simple contact ou effleurement. Elle est exacerbée par le froid. Un homme âgé de 39 ans, droitier, technicien en réparation électronique, sans antécédents pathologiques particuliers ; se présentant avec une douleur paroxystique et exquise de l'index de la main droit, évoluant depuis deux ans. À l'examen on note une zone pulpaire très sensible à la pression, avec absence de signes objectifs locaux. La radiographie standard réalisée n'objective pas de lésions osseuses. L'IRM datant de 1 an était normale. Une deuxième IRM dans un intervalle d'un an a été réalisée, et a montré un aspect évocateur d'une tumeur glomique sous forme d'un hyposignal en T1 (A), suivi d'un hypersignal en T2 (B) Une exérèse chirurgicale par voie pulpaire direct de la tumeur a été réalisé, l'aspect per opératoire était très évocateur : la tumeur de 3 mm d'aspect bleuté était bien limitée encapsulée (C), et l'examen histologique de la pièce a confirmé le diagnostic. Les suites opératoires ont été simples, après 1 an de recul, le patient est asymptomatique. Les tumeurs glomiques sont rares, mais elles peuvent siégées dans tout le corps. Ce sont des malformations hamartomateuses du glomus normal. Les tumeurs peuvent être non palpables parce qu'ils sont très petites et profondes.Cette affection est rare, avec un siège de prédilection sous-unguéal. La triade diagnostic classique se compose de douleur spontanée, a la pression, et l'hypersensibilité au froid. L'évolution est lente et le retard diagnostique est fréquent. Les examens complémentaires qui ont été utilisés avec un succès variable pour le diagnostic des TG sont: la transillumination, l'artériographie, la thermographie, l'échographie avec séquence doppler, et la scintigraphie. l'IRM est considéré comme le test diagnostique le plus sensible. Le traitement chirurgical est curatif et la récidive est rare.

**Figure 1:**
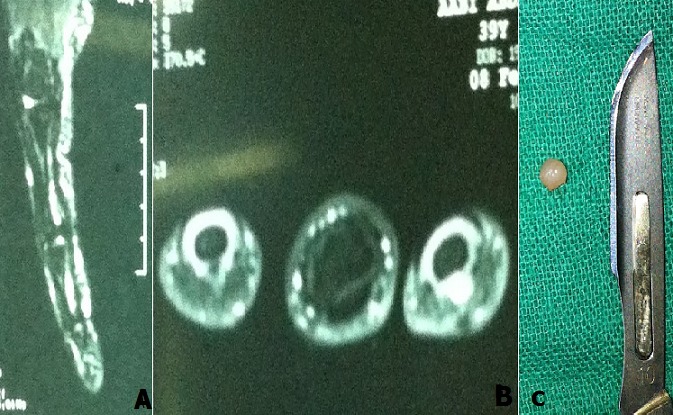
A) un aspect IRM évocateur d’une tumeur glomique sous forme d’un hyposignal en T1; (B) hypersignal en T2; C) la tumeur glomique

